# Neurobiological origins of individual differences in mathematical ability

**DOI:** 10.1371/journal.pbio.3000871

**Published:** 2020-10-22

**Authors:** Michael A. Skeide, Katharina Wehrmann, Zahra Emami, Holger Kirsten, Annette M. Hartmann, Dan Rujescu, Legascreen Consortium

**Affiliations:** 1 Department of Neuropsychology, Max Planck Institute for Human Cognitive and Brain Sciences, Leipzig, Germany; 2 Institute of Psychology, Humboldt University of Berlin, Berlin, Germany; 3 Department of Psychiatry, University of Bern, Bern, Switzerland; 4 The Hospital for Sick Children, Toronto, Canada; 5 Institute for Medical Informatics, Statistics and Epidemiology, University of Leipzig, Leipzig, Germany; 6 Department of Psychiatry, Psychotherapy and Psychosomatics, Martin-Luther-University Halle-Wittenberg, Halle (Saale), Germany; Universität Tübingen, GERMANY

## Abstract

Mathematical ability is heritable and related to several genes expressing proteins in the brain. It is unknown, however, which intermediate neural phenotypes could explain how these genes relate to mathematical ability. Here, we examined genetic effects on cerebral cortical volume of 3–6-year-old children without mathematical training to predict mathematical ability in school at 7–9 years of age. To this end, we followed an exploration sample (*n* = 101) and an independent replication sample (*n* = 77). We found that *ROBO1*, a gene known to regulate prenatal growth of cerebral cortical layers, is associated with the volume of the right parietal cortex, a key region for quantity representation. Individual volume differences in this region predicted up to a fifth of the behavioral variance in mathematical ability. Our findings indicate that a fundamental genetic component of the quantity processing system is rooted in the early development of the parietal cortex.

## Abstract 2

How does genetic variation give rise to differences in mathematical ability? Combining genotyping with brain imaging in children, this study shows that math performance can be predicted before school entry from individual volume differences of the right parietal cortex that are associated with variations in the cortical growth gene ROBO1.

## Introduction

Mathematical ability is shaped by a complex interplay between genetic and environmental factors, in which genetic variance explains about 60% of the behavioral variance [1]. Building on this evidence, several DNA variants have been found to be associated with mathematical performance, including *RP11-815M8*.*1*, *FLJ20160*, *ROBO1*, *FAM43A/LSG1*, *SFT2D1*, *DLD*, *NRCAM*, *NUAK1*, *C14orf64*, and *GRIK1* [2–5]. Many of these variants are located on genes that also express proteins in nerve cell tissue [6]. Little is known, however, about how expression patterns of math-related genes are distributed over the developing human brain (www.brainspan.org). Accordingly, it is an open question how the developing brain as an intermediate phenotype might bridge the gap from genetic variability to mathematical ability.

Mathematical cognition draws on diverse, dynamically interacting neural systems [7]. Beyond visual and/or auditory machinery, essential processing resources are provided by attention and execution systems of the prefrontal cortex, a premotor short-term memory rehearsal mechanism, a long-term memory storage unit in the medial temporal lobe, and, most specifically, the parietal cortex, which builds visuospatial quantity representations [7–11]. All these anatomically broadly distributed systems could thus be related to previously reported genes linked to mathematical ability.

The aim of the present study was to explore associations between known math candidate genes and brain structure in young children that had not yet received math instruction. Furthermore, we investigated longitudinally whether these associations would predict mathematical performance in school. Targeting a structural magnetic resonance imaging measure (i.e., grey matter volume) was motivated by the currently available neurobiological data for math candidate genes. These data provide converging evidence that math-related genes play a role for grey matter growth, in particular synapse formation, intracortical axon branching, and neuronal migration [12–14]. Importantly, the rationale behind focusing on an initially unschooled sample was to capture potential neurobiological predispositions, not consequences, of individual mathematical learning success.

As a first step of our analysis, we selected 18 single nucleotide polymorphisms (SNPs) on 10 genes previously found to be significantly associated with mathematical performance. Associations between these SNPs and grey matter volume were then calculated at the whole-brain level in an exploration sample (*n* = 101) and, guided by power analyses, in an independent replication sample (*n* = 77) of 3–6-year-old children. To this end, we used a multivariate statistical model quantifying joint effects of SNPs located in the same gene. Specifically, we quantified the associations among all SNPs and then related the resulting covariance matrix to the MRI data matrix. We did not apply a predefined coefficient-of-determination or *p*-value threshold to preselect particular SNPs. This approach has been shown to detect biologically valid dependencies between SNPs and to increase statistical power compared to classical univariate approaches [15]. Finally, within the volumetric clusters obtained from the genetic association model, we ran multivariate searchlight analyses to decode voxels that are related to individual math test scores in second grade (7–9 years of age).

Following the current state of knowledge about neural systems contributing to mathematical cognition, we hypothesized that significant association and prediction effects could be expected in prefrontal, premotor, medial temporal, and inferior parietal cortices.

## Results

### Descriptive participant data

Genotypes and structural brain scans were acquired at 3–6 years in an exploration sample (*n* = 101) and a replication sample (*n* = 77). Standardized age-normed test scores of mathematical ability collected at 7–9 years were available for *n* = 84 out of 101 children in the exploration sample and for *n* = 75 out of 77 children in the replication sample. Demographic features and behavioral test performance did not differ significantly between those children that completed both waves of data collection and those children that dropped out after the first wave of data collection (exploration sample: all *z* < 2, all χ < 1, all *P* > 0.05; replication sample: all *z* < 2, all χ < 1, all *P* > 0.05). Sample characteristics based on complete datasets are specified in Table 1.

**Table 1:** Demographic information and behavioral test performance.

	Exploration sample	Replication sample	Comparison
**Age**^1^ (mean ± SD^2^, min–max)	4.88 ± 0.98, 3.08–6.17	4.04 ± 0.56, 3.16–5.08	*z* = 10.95, *P* < 0.001^6^
**Sex** (male/female)	46/38	38/39	χ(1) = 0.27, *P* = 0.602^7^
**Handedness** (right, left, ambidextrous)	78, 3, 3	72, 0, 5	χ(1) = 141.94, *P* < 0.001^7^
**Total intracranial volume**^3^ (mean ± SD, min–max)	1453 ± 128, 1083–1772	1438 ± 112, 1060–1733	*z* = 0.85, *P* = 0.398^6^
**Maternal education**^4^ (mean ± SD, min–max)	4.42 ± 1.25, 2–7	4.87 ± 1.19, 3–6	*z* = 2.38, *P* = 0.017^6^
**Nonverbal IQ** (mean ± SD, min–max)	103 ± 15, 70–139	101 ± 11, 77–126	*z* = 0.84, *P* = 0.402^6^
**Mathematical ability**^5^ (mean ± SD, min–max)	58 ± 32, 1–100	57 ± 28, 2–100	*z* = 1.04, *P* = 0.297^6^

^1^Age in years at which children underwent structural magnetic resonance imaging

^2^Standard deviation

^3^in cm^3^

^4^0–7 Likert scale (see Methods for details)

^5^Percentile ranks

^6^Mann–Whitney *U* tests

^7^Pearson χ^2^ tests

### Associations between math candidate genes and grey matter volume at age 3–6 years

**Fig 1:**
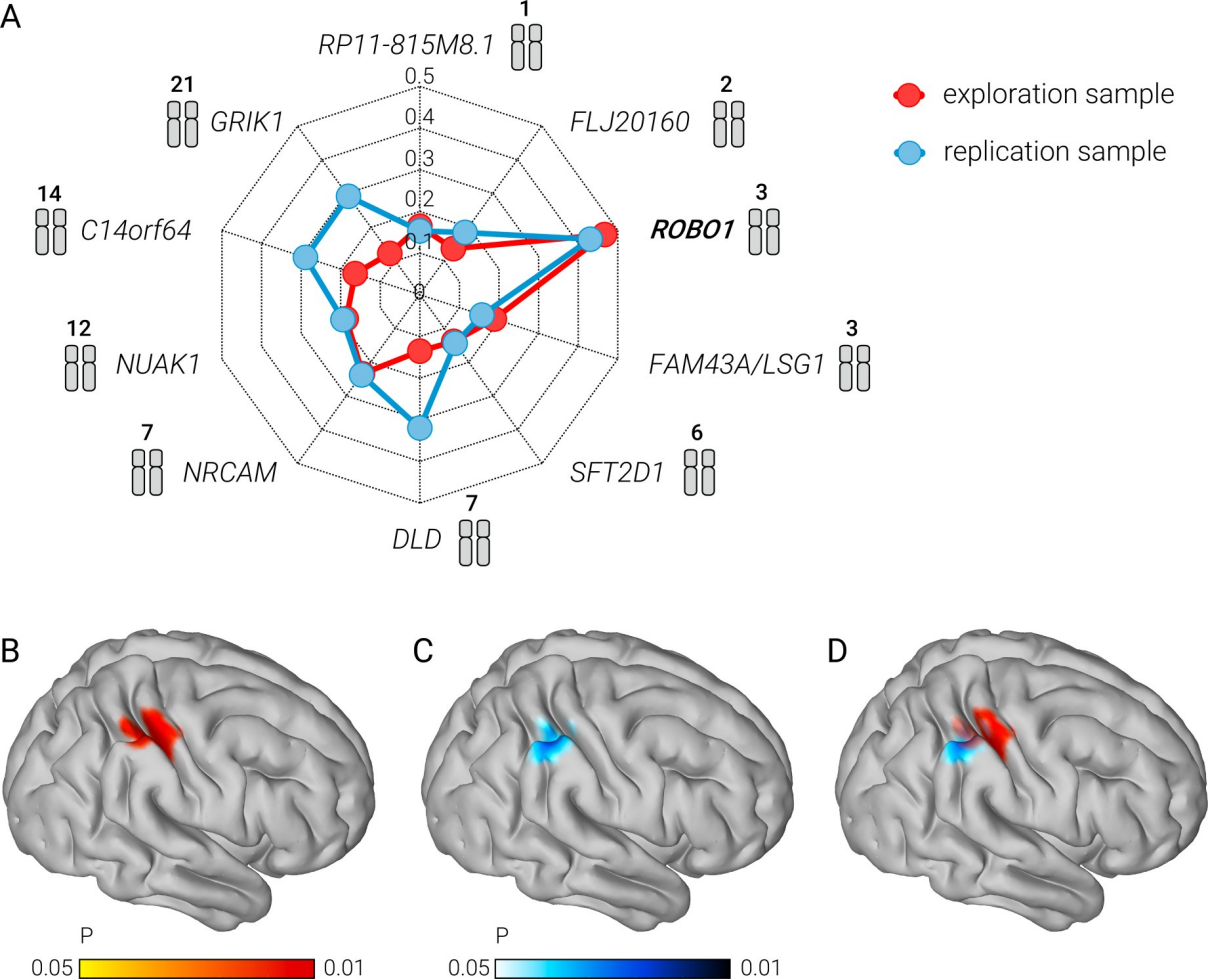
Grey matter volume of the right parietal cortex of 3-6-year-old children without mathematical training is associated with the cortical growth gene *ROBO1*. (A) Outer corner points depict 10 known math candidate genes and their corresponding numbered chromosomes. Dashed lines depict *R^2^* statistics quantifying the strength of associations between the genes and the grey matter volume images. The further away a point is from the center, the stronger the association is. Orange points/lines refer to the exploration sample, and blue points/lines refer to the replication sample. (B–D) Right sagittal view on a cortical surface projection of *P*-value images showing the right parietal clusters that were significantly associated with *ROBO1*. Results are shown separately for the exploration sample (B), the replication sample (C), and the overlap between both samples (D). The color bar indicates the range of *P*-values with a lower threshold of *P* < 0.05 and an upper threshold of *P* < 0.01, family-wise-error-corrected for the number of voxels and genes tested. The numerical data used in this figure are included in S1 Data.

In the exploration sample, a significant association at a threshold of *P* < 0.05 (family-wise-error-corrected for the number of voxels and genes tested) was detected for the gene *ROBO1* (max. *R^2^* = 0.47) (Fig 1A), but none of the other nine genes tested (Table 2). The achieved power to detect this large effect was 0.94. The sample size needed to replicate this effect with a power of 0.8 was *n* = 71. Accordingly, in an independent sample of *n* = 77, the association effect of *ROBO1* was replicated testing the same set of genes (Table 2) at the same statistical threshold (max. *R^2^* = 0.43) (Fig 1A). The effects of age, sex, handedness, and total intracranial volume were controlled in the models.

**Table 2:** Genotypic information.

		SNP^2^			Participants per genotype^4^			Genotypes		
Chrom^1^	Gene	Proxy	MAF^3^^,^^4^	HWE^4^^,^^5^	HoMa^6^	Het^7^	HoMi^8^	HoMa^6^	Het^7^	HoMi^8^
1	*RP11-815M8*.*1*^9^	rs12130910 rs6604676^12^	0.35 0.34	0.66 0.61	40 35	49 32	11 10	GG	GA	AA
2	*FLJ20160*^10^	rs12613365 rs3811609^13^	0.28 0.25	0.22 0.55	50 44	46 27	5 6	GG	TG	TT
3	*ROBO1*^11^	rs162870	0.38 0.34	0.67 0.45	15 7	44 38	38 32	CC	AC	AA
3	*ROBO1*^11^	rs331142	0.26 0.26	0.29 0.37	58 44	34 26	9 7	TT	GT	GG
3	*ROBO1*^11^	rs12495133	0.37 0.38	0.29 0.63	43 28	42 39	16 10	CC	CA	AA
3	*ROBO1*^11^	rs11127636	0.42 0.44	0.21 0.65	20 16	41 36	36 25	CC	AC	AA
3	*ROBO1*^11^	rs4535189	0.44 0.47	1 0.65	31 20	49 41	20 16	TT	TC	CC
3	*ROBO1*^11^	rs7614913	0.44 0.31	0.54 0.79	34 37	46 32	21 8	TT	TC	CC
3	*ROBO1*^11^	rs6548628	0.45 0.49	1 0.25	20 23	50 33	31 21	CC	AC	AA
3	*ROBO1*^11^	rs9853895	0.50 0.40	0.32 0.48	28 14	45 34	28 29	TT	CT	CC
3	*ROBO1*^11^	rs1995402	0.40 0.39	0.30 0.63	39 30	43 34	19 13	CC	CA	AA
3	*FAM43A/LSG1*^9^	rs789859 rs4677854^14^	0.41 0.40	0.1 0.47	40 26	40 41	21 10	GG	TG	TT
6	*SFT2D1*^9^	rs4144887 rs4144886^12^	0.18 0.27	0.3 1	70 41	26 31	5 5	CC	CT	TT
7	*DLD*^10^	rs6947045 rs886774^15^	0.41 0.40	0.41 1	38 27	44 38	19 12	GG	GA	AA
7	*NRCAM*^10^	rs2300052 rs13245242^12^	0.25 0.23	0.6 0.54	55 46	40 26	5 5	GG	GA	AA
12	*NUAK1*^10^	rs1215603 rs2913132^16^	0.41 0.40	0.3 0.48	32 29	54 34	14 14	CC	TC	TT
14	*C14orf64*^9^	rs2809115 rs9646139^17^	0.46 0.42	0.55 1	31 26	47 37	23 14	GG	GA	AA
21	*GRIK1*^10^	rs363449 rs9978417^18^	0.40 0.38	0.15 0.004	33 23	56 49	12 5	TT	TC	CC

^1^Chromosome

^2^Single nucleotide polymorphism

^3^Minor allele frequency

^4^First line: exploration sample, Second line: replication sample

^5^Hardy–Weinberg Equilibrium

^6^Homozygous major allele

^7^Heterozygous alleles

^8^Homozygous minor allele

^9^ [3]

^10^ [2]

^11^ [4]

^12^*R*^*2*^ = 1

^13^*R*^*2*^ = 0.98

^14^*R*^*2*^ = 0.58

^15^*R*^*2*^ = 0.86

^16^*R*^*2*^ = 0.99

^17^*R*^*2*^ = 0.88

^18^*R*^*2*^ = 0.95

Associations at the whole-brain level were considered significant when local clusters remained under an arbitrarily defined height threshold of *P* < 0.05 (family-wise-error-corrected for the number of voxels and genes tested) and exceeded an arbitrary extent threshold of k > 100 voxels. In the exploration sample, the effect of *ROBO1* was localized in the right parietal cortex encompassing the dorsal lip of the intraparietal sulcus extending into the adjacent gyrus of the ventral superior parietal lobule (peak MNI coordinates: +45–33 +57; k = 437 voxels) (Fig 1B). No other areas exceeded a spatial extent threshold of k = 100 voxels. In the replication sample, the effect of *ROBO1* was also localized in the right parietal cortex (peak MNI coordinates: +49–41 +55; k = 304 voxels) where both clusters overlapped (Fig 1C and 1D).

### Prediction of math performance at age 7–9 years from parietal grey matter volume at age 3–6 years

**Fig 2:**
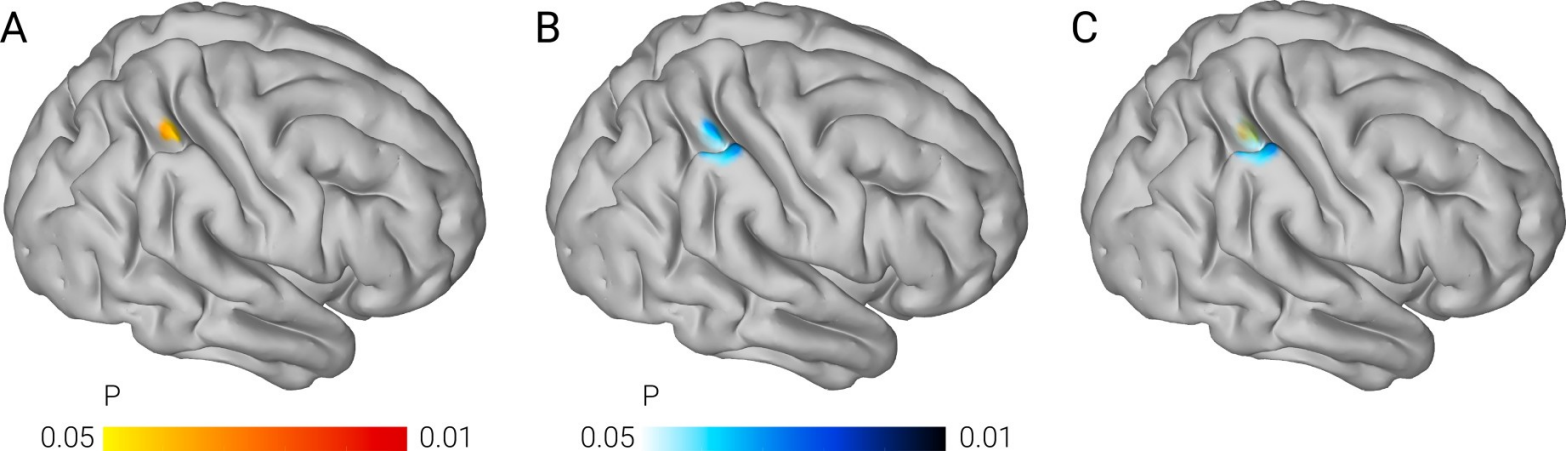
*ROBO1*-associated grey matter volume of the right parietal cortex of 3–6-year-old children without mathematical training predicts mathematical ability in school at 7–9 years of age. (A–C) Right sagittal view on a cortical surface projection of *P*-value images showing voxels within parietal clusters that were significantly associated with *ROBO1* at 3–6 years of age and with individual scores of a mathematical ability test conducted at 7–9 years of age. Results are shown separately for the exploration sample (A), the replication sample (B), and the overlap between both samples (C). The color bar indicates the range of *P*-values with a lower threshold of *P* < 0.05 and an upper threshold of *P* < 0.01 (voxel-wise permutation-corrected). The numerical data used in this figure are included in S2 Data.

Individual grey matter volume within the right parietal cluster that was associated with *ROBO1* at 3–6 years of age was significantly associated with individual scores in a comprehensive behavioral math test taken at 7–9 years of age. Associations were significant at a threshold of *P* < 0.05 (permutation test corrected for the number of voxels tested) in the exploration sample (max. *R*^*2*^ = 0.10) (Fig 2A) and the replication sample (max. *R*^*2*^ = 0.22) (Fig 2B) in an overlapping part of the right parietal cortex (Fig 2C). Additional separate brain-behavior association analyses revealed no evidence for a dissociation between numeracy and calculation skills in the exploration sample (*z* = 0.23, *P* = 0.410) and the replication sample (*z* = 0.40, *P* = 0.343). The effects of age, sex, handedness, total intracranial volume, maternal education, and nonverbal IQ were controlled in the models.

## Discussion

In this study, we explored associations between 18 SNPs on 10 math candidate genes and whole-brain grey matter volume in an exploration sample of 101 and a replication sample of 77 unschooled children aged 3–6 years. We observed that the gene *ROBO1* was significantly associated with grey matter volume in dorsal parts of the right intraparietal sulcus and ventral parts of the right superior parietal lobule. Grey matter volume patterns within these regions revealed significant associations with math test scores at 7–9 years of age in second grade.

### Role of *ROBO1* for grey matter development

The reported link between *ROBO1* and grey matter volume is supported by a large body of molecular genetic literature suggesting that this gene plays a crucial role for prenatal growth of the rodent neocortex. Specifically, there is converging evidence that *ROBO1* regulates neuronal migration (i.e., the positioning of neurons in cortical layers during intrauterine brain maturation) [13, 16, 17]. Moreover, *ROBO1* might also contribute to the proliferation of neurons in the cortex [18].

Human gene expression data collected ex vivo corroborate the neuroanatomical validity of the effect that we detected here in vivo. This work demonstrates that the proteins encoded by *ROBO1* are consistently expressed in the parietal cortex of two 3-year-old and two 8-year-old children (www.brainspan.org). Our results do not, however, allow us to draw any firm conclusions about negative findings in other areas.

### *ROBO1*, the parietal cortex, and mathematical ability

The current findings suggest that individual differences in right parietal cortex growth might be an intermediate phenotype filling the explanatory gap in previously reported associations between DNA variation and behavioral mathematical performance. This interpretation is compatible with numerous studies showing that the parietal cortex specifically contributes to mathematical cognition from childhood on and keeps this decisive role in adulthood [19, 20]. In particular, the intraparietal sulcus and the superior parietal lobule provide the neural resources for quantity detection, which remains an essential basic component even for higher-order mathematical problem solving [20].

Interestingly, nonsymbolic quantity processing skills typically emerge in the first months of life without formal education and exhibit marked individual differences from the onset of ontogeny [21]. Following the results of the present study, we generate the working hypothesis that even these very early differences might already be explained by differences in right parietal cortex volume, which are related to *ROBO1* variability. Further experiments with infants are needed to confirm this hypothesis.

### Hemispheric specialization of the parietal cortex

In the adult brain, functional activation during mathematical processing is consistently seen in bilateral parietal cortices [22, 23]. In contrast, and in line with our structural findings, children more strongly recruit the right (compared to the left) parietal cortex when performing mathematical tasks, according to a recent meta-analysis [24]. A deeper understanding of this developmental difference, which presumably disappears with further experience, remains as a future challenge.

### Conclusion

Our study indicates that up to a fifth of variance in mathematical ability can be predicted from early individual differences in right parietal cortex volume which is related to the cortical growth gene *ROBO1*. These results suggest that genetic variability might shape mathematical ability by sculpting the early development of the brain’s basic quantity processing system.

## Methods

### Participants

Participants were recruited between 2012 and 2013, mainly from the Leipzig metropolitan area but also from other parts of Germany. We incentivized families to take part in the study by telling them that the current study would improve our understanding of the origins of developmental learning disorders. All parents or guardians gave written informed consent and all children gave verbal informed consent to participate. Participation was rewarded with a “junior researcher diary” and a small educational gift for each child and a reimbursement for the MRI scanning session (€15) and the behavioral assessment sessions (€7.50). The study was approved by the Ethics Committee of the University of Leipzig, Germany (approval number 320-11-26092011). Participants were excluded from further analysis if they (A) had a history of neurological and/or psychiatric disorders, (B) had hearing and/or vision disorders, (C) had attention deficit hyperactivity disorder, (D) scored more than two standard deviations (SDs) below the age average in a nonverbal IQ test and thus met a criterion for mental retardation, (E) did not comply with the experimental procedures in a training session, and/or (F) moved in the scanner so that data quality was compromised. Data were collected between 2012 and 2019. All of these procedures are in compliance with the relevant ethical regulations specified in the Declaration of Helsinki.

### Genotyping

DNA from saliva was extracted using standard procedures as described in [25] or using Oragene DNA Genotek Kits (Kanata, Ontario, Canada). In the exploration sample, genotyping for all SNPs but rs331142, rs12495133, and rs1995402 was performed with the bead chip Infinium HumanCoreExome Psych Chip. Bead chip genotyping was carried out according to the manufacturer’s instructions and was analyzed using Illumina’s GenomeStudio Genotyping Module. Variants rs331142, rs12495133, and rs1995402 were genotyped via matrix-assisted laser desorption/ionization time-of-flight mass spectrometry (iPLEX, Agena, Hamburg, Germany). We observed a high concordance rate for additional SNPs genotyped with both technologies (99.99%). In the replication sample, all variants were genotyped via matrix-assisted laser desorption/ionization time-of-flight mass spectrometry (iPLEX, Agena, Hamburg, Germany).

Genotyping data had to fulfill the following quality measures: SNP-wise exact Hardy–Weinberg Equilibrium (HWE) *P* > 0.05 [26], SNP-wise call rate > 95%, individual-wise call rate > 90%, MAF >0.05, and 100% fit between genotypes of individuals that were measured in duplicates. One variant (rs363449) with deviation from the HWE (*p* = 0.004) was included, as there was no mismatch between genotyped Central European trios (Coriell Institute for Medical Research, Camden, New Jersey, United States of America) and the HapMap database (https://www.ncbi.nlm.nih.gov/probe/docs/projhapmap/).

SNPs not directly covered by genotyping were substituted by an appropriate proxy revealing the highest linkages disequilibrium (*R*^*2*^) with the original SNP (Table 2) using 1000 Genomes version 1 phase 3 as reference panel [27]. It should be noted as a limitation that rs4677854 cannot be considered as a good proxy for rs789859 given the *R*^*2*^ of 0.58.

Relatedness among the analyzed participants was assessed by analyzing kinship (IBS) measures between participants using R and GenABEL (28). We identified five siblings (expected IBS = 0.5) using the conventional cutoff of 0.354 (the geometric mean of 0.5 and 0.25) and one first-cousin pair (expected IBS = 0.125) using the conventional cutoff of 0.088 (calculated accordingly), while the remaining participants were unrelated (IBS ≤ 0.088). Accordingly, we also ran the gene–brain association analysis without five siblings and one cousin. This reanalysis reproduced the identical peak MNI coordinates (+45–33 +57) and statistics in all 437 voxels.

### MRI data acquisition and preprocessing

T1-weighted three-dimensional magnetization-prepared rapid-acquisition gradient echo (MP2RAGE) images [29] were acquired on a 3.0-Tesla Siemens TIM Trio whole-body magnetic resonance scanner using a 12-radiofrequency-channel head coil and the following parameters: TR = 5,000 ms, TE = 2.82 ms, TI_1_ = 700 ms, TI_2_ = 2,500 ms, FOV = 256 x 240, matrix size = 250 x 219 x 144 and voxel size = 1.3 x 1.3 x 1.3 mm^3^.

Image quality was assessed in a two-step procedure. In the first step, we made sure by visual inspection that each image was free of artifacts and/or anatomical abnormalities. In the final step, image quality was evaluated automatically quantifying noise and inhomogeneity using the Computational Anatomy Toolbox (CAT) (http://dbm.neuro.uni-jena.de/cat) implemented in the Statistical Parametric Mapping 12 (SPM 12) software (http://fil.ion.ucl.ac.uk/spm/). Only images with a rating of at least 80 (indicating good quality) were retained for further analysis.

Grey matter volume images were computed by running a voxel-based morphometry analysis in CAT and SPM 12. To this end, we first created a customized multitissue probability map (including grey matter, white matter, cerebrospinal fluid, bone, soft tissue, and air/background) with the Template-O-Matic Toolbox (https://irc.cchmc.org/software/tom.php) using the dataset acquired during the NIH MRI study of normal brain development as the data basis. This map matched the age and sex of the present sample and served as a prior to compute a sample-specific template in Montreal Neurological Institute (MNI) space using the Diffeomorphic Anatomical Registration Through Exponentiated Lie Algebra algorithm. Next, we normalized each individual T1-weighted image to the sample-specific template and segmented it into grey matter, white matter, cerebrospinal fluid, dura, soft tissue, and air. Based on these data, we were able to estimate the total intracranial volume. Grey matter volume images were then calculated while modulating for nonlinear effects to preserve local volumetric values. These images were finally smoothed with an 8-mm full-width at half-maximum Gaussian kernel.

### Maternal education data

Maternal education was assessed with a customized in-house questionnaire and defined as the sum of school education and professional education. School education was quantified on a scale from 0 to 3 (0 = no school graduation, 1 = graduation after 9 years (German “Hauptschulabschluss”), 2 = graduation after 10 years (German “Mittlere Reife”), 3 = high school graduation). Higher education was quantified on a scale from 0 to 4 (0 = no professional degree, 1 = vocational degree, 2 = university of applied sciences degree, 3 = college graduate, 4 = graduate degree). According to this scale, an index of 4.5 would represent an average maternal education level. The mean index values of the current samples (exploration sample: 4.42, replication sample: 4.87) thus indicate average maternal education levels ranging from poorly educated to highly educated mothers (index values 2–7).

### Behavioral testing

Handedness was measured with a customized in-house test, in which children were asked to perform or simulate everyday activities with their hands so that we could calculate a laterality quotient (LQ). Right-handedness was defined as LQ > +48, left-handedness as LQ ≤ –28, and ambidexterity as –28 < LQ ≤ +48.

The Perceptual Reasoning subscale of the Wechsler Intelligence Scale for Children (WISC-IV) was used to derive a nonverbal IQ score (https://www.testzentrale.de/shop/wechsler-intelligence-scale-for-children-deutsche-ausgabe-fourth-edition.html).

Mathematical ability was assessed using the Heidelberg Arithmetic Test (https://www.testzentrale.de/shop/heidelberger-rechentest.html). This comprehensive test instrument consists of 11 subtests covering addition, subtraction, multiplication, division, symbolic and nonsymbolic quantity comparison, quantity estimation, numerical sequencing, and counting. Correct answers were added together and transformed into a percentile rank based on age norms for three subscales: numeracy, calculation, and total mathematical ability.

Handedness and intelligence were assessed individually in a single session in a small child laboratory room. Mathematical ability was assessed as a group test (max. 15 children) in a separate session in a larger seminar room. In each sample, these data were acquired by a maximum of three different research assistants that were thoroughly familiarized with the testing procedure beforehand. Before collecting the data, each assistant passed three supervised practice sessions with children that were not enrolled in the current study.

### Gene–brain association analysis

A multilocus model based on least-squares kernel machines was combined with conservative voxel-wise statistical inference based on the random field theory to test for joint nonlinear associations between 18 SNPs and multivariate patterns in grey matter volume images while removing the linear effect of the covariates’ age, sex, handedness, and total intracranial volume [15]. The resulting *R*^*2*^ statistic images were tested for significance using a permutation procedure (running 10,000 permutations) based on parametric tail approximation and subsequently transformed to *P*-value images [15]. Associations were considered significant when clusters remained under an arbitrarily defined height threshold of *P* < 0.05 (family-wise-error-corrected for the number of voxels and genes tested) and exceeded an arbitrary extent threshold of k > 100 voxels. During the family-wise-error-correction, the statistical threshold of each voxel was adjusted by (1) multiplying it with the total number of 408,965 voxels tested while taking into account the effective smoothness of the signals and then (2) multiplying the resulting threshold of each voxel with the total number of 10 genes tested. These analyses were run in Matlab (https://www.mathworks.com) and SPM 12.

Power analyses were conducted using the G*Power toolbox (http://www.gpower.hhu.de). The achieved power was calculated post hoc using the statistical framework of a goodness of fit test based on the observation that the multivariate model we applied produces an approximate χ^2^ test statistic that can similarly be converted into a correlation coefficient and a *p*-value [15]. The input parameters of this power calculation were the effect size of Cohen’s *w* = 0.47, the alpha error probability of 0.05, and the sample size of *n* = 101. The sample size needed to replicate this effect with a power of 0.8 was calculated a priori also within the framework of a χ^2^ goodness of fit test using the effect size of Cohen’s *w* = 0.47, the alpha error probability of 0.05, and the power level of 0.8 as the input parameters.

### Brain–behavior association analysis

A searchlight-based multivariate pattern analysis approach was used to identify voxels that were significantly associated with math test scores within the clusters derived from the genetic association analyses. To this end, for each voxel within these clusters, we defined a spherical, 4-mm surrounding region (the searchlight) and performed support vector regression analyses for each possible searchlight position within a 10-fold cross validation design. Coefficients of determination (*R*^*2*^) were assigned to each voxel at its center and nonparametrically assessed for significance by running 10,000 permutations of the training and test data to yield a voxel-wise null distribution. During the permutation-test correction for false positives, the observed results were randomly resampled 10,000 times to build an empirical estimate of the null distribution to draw the test statistic (coefficient of determination) from. Voxels were identified as significant by counting the number of times the test statistic was smaller or greater than the statistic value obtained from the permuted data sets and multiplying this value by the minimal *P*-value of the permutation test (1/(n+1), *n* = 10,000). Effects of covariates of no interest, including age, sex, handedness, total intracranial volume, maternal education level, and nonverbal IQ were removed, based on a cross-validated confound regression method [30]. The analyses were carried out using The Decoding Toolbox (https://sites.google.com/site/tdtdecodingtoolbox/) and Matlab. Coefficients of determination of the separate brain-behavior association analyses for numeracy and calculation skills were compared by running Meng’s z-tests.

## Supporting information

S1 Data Numerical values (*R*^*2*^ coefficients) related to the radar plot in Fig 1. (XLSX)

S2 Data Numerical values (*p*-values) related to the brain image rendering in Fig 2. (XLSX)

## Abbreviations

CAT: Computational Anatomy Toolbox
HWE: Hardy–Weinberg Equilibrium
LQ: laterality quotient
MAF: minor allele frequency
MNI: Montreal Neurological Institute
MP2RAGE: magnetization-prepared rapid-acquisition gradient echo
SD: standard deviation
SNP: single nucleotide polymorphism
SPM 12: Statistical Parametric Mapping 12
WISC-IV: Wechsler Intelligence Scale for Children
